# Case Report Presentation of Ultrasound-guided Erector Spinae Plane Block in Shoulder Surgery: Three Patients and Two Different Results

**DOI:** 10.7759/cureus.3538

**Published:** 2018-11-03

**Authors:** Onur Selvi, Serkan Tulgar, Zeliha Ozer

**Affiliations:** 1 Aneasthesiology, Maltepe University Faculty of Medicine, Istanbul, TUR; 2 Anaesthesiology, Maltepe University Faculty of Medicine, Istanbul, TUR

**Keywords:** regional anaesthesia, orthopaedic surgery

## Abstract

Erector spinae block (ESPB) is an effective therapy for chronic shoulder pain. However, ESPB has not been used as a postoperative analgesia method in shoulder surgeries. In this case report, we report three patients undergoing shoulder surgeries that received ESPB preoperatively for postoperative analgesia. All patients had relief of preoperative pain and no associated motor block. Two of the patients manifested with low maximum pain scores (4/10, 3/10) on a numeric rating scale (NRS). The other patient reported a maximum pain score of 8/10 on NRS. While this patient’s shoulder mobility immediately improved after ESPB application, the ESPB did not provide adequate analgesia for the postoperative period. The use of the ESPB for acute postoperative analgesia after shoulder surgery is novel and clinically interesting. However, postoperative analgesia was not completely opioid-sparing. Consequently, the efficiency of ESPB at the level of T2 for postoperative analgesia should be considered for surgeries that involve the shoulder cap given the possible inadequate migration of local anesthetic into the cervical plexus. Clinicians should carefully consider an ESPB as a postoperative analgesic option when considering shoulder operations and the possibility for the incomplete spread of local anesthetic in targeted neural structures.

## Introduction

Erector spinae plane block (ESPB) has been reported for a variety of indications [1-2]. ESPB has been successfully used to treat chronic shoulder pain [3], and the local anesthetic spread was reported to reach the level of C3 when it was performed at T2 [4]. Having achieved shoulder analgesia after applying ESPB as high as T2, we were encouraged to use it for postoperative pain treatment in arthroscopic shoulder surgery—an operation that can cause severe pain [5]. To our knowledge, this is the first description of the use of ESPB in shoulder surgery. Herein we present three case reports of ESPB applied in two patients undergoing arthroscopic shoulder surgeries for chronic shoulder pain and in one patient with a Neer Type 3 proximal humeral fracture.

Written informed consent was obtained from all patients in this report. Ethics board approval for case reports is not required by our institute.

## Case presentation

All of the patients received identical protocols for the induction and maintenance of general anesthesia, application of ESPB, preoperative and postoperative analgesia, and patient-controlled analgesia (PCA). Intravenous (IV) midazolam (2 mg) was given for sedation. After providing standard basic anaesthetic monitoring, each patient was placed in a sitting position. A 10-MHz ultrasound device (Mindray 9900 Plus, Mindray, Hamburg, Germany) was used, and the T2 transverse process was selected as the needle insertion point. The ultrasound probe was placed 3 cm lateral to the T2 spinous process in a longitudinal parasagittal orientation to visualise the transverse process. Under sterile conditions, a 5-cm, 21-gauge needle (BRAUN Stimuplex A, Germany) was inserted using the out-of-plane technique parallel to the sagittal plane directly over the transverse process. We confirmed the location by visualization of hydrodissection with 0.9% NaCl. We injected 25 mL 0.375% bupivacaine with 5 mL of 2% lidocaine and observed the linear spread of the local anaesthetic over the transverse process. Following injection, all patients expressed sudden pain relief in their shoulders and increased mobility. Induction of anesthesia was completed with the patients in a supine position with propofol (2 mg/kg), fentanyl (100 µg), and rocuronium bromide (0.6 mg/kg). General anesthesia was maintained with remifentanil infusion at the rate of 0.08 µg/kg/minute and 0.6 minimum alveolar concentration sevoflurane as the inhaled agent. For postoperative analgesia, all patients received 1 g paracetamol and 20 mg tenoxicam via IV administration. In the postanesthesia care unit (PACU), 25 µg fentanyl was applied if the patient reported a pain score of 4/10 or higher on a numeric rating scale (NRS). In case of pain persisting longer than 20 minutes, an additional 25 µg fentanyl was added to the treatment. For postoperative analgesia, all patients were scheduled to receive 1 g paracetamol via IV administration every eight hours. As rescue analgesia (i.e., if the patient-reported pain score exceeded 4/10 on NRS), diclofenac sodium (75 mg) was administered intramuscularly (IM). If the pain remained at the same level after a further hour had passed, IV meperidine (50 mg) was added. PCA with tramadol at 3 mg/mL concentration was programmed with no basal infusion; the demand dose was 10 mg with a 20-minute lock-out interval and was started in the PACU. Patients were closely monitored by a trained nurse, and a PCA follow-up form was completed in 24 hours.

Patient One was a 30-year-old woman, American Society of Anesthesiologists (ASA) Classification I (weight, 64 kg; height, 168 cm) with no coexisting diseases. She was scheduled for arthroscopic shoulder surgery. Her preoperative shoulder mobility over the shoulder joint was limited to 120° with severe pain. The patient described acute relief of the pain in her shoulder and increased mobility after ESPB performed before the surgery started. She was treated surgically for arthroscopic acromioplasty and bursectomy. Her pain was recorded as 1/10 on NRS and needed no additional analgesia in the PACU. Her maximum dynamic pain score was 4/10 on NRS. Her 24-hour PCA tramadol consumption was 40 mg total.

Patient Two was a 65-year-old woman, ASA Class I (weight, 55 kg; height 165 cm) registered for a Neer Type 3 humeral fracture and surgical repair. She reported sudden pain relief after the application of ESPB. An open reduction and internal fixation via the anterolateral method was performed under general anaesthesia. In the PACU, she reported her pain as 2/10 on NRS with mobilization. At the 24-hour follow-up evaluation, she reported no pain score higher than 3/10 on NRS, and no rescue analgesic treatment was administered. Her total PCA consumption was 240 mg in 24 hours. Although her PCA consumption was high, she needed no fentanyl in the PACU nor additional diclofenac sodium and meperidine as rescue analgesia.

Patient Three was a 39-year-old woman, ASA Class I (weight, 55 kg; height, 165 cm) who presented for arthroscopic shoulder surgery. She had severe pain (8/10) on NRS with abduction-adduction at the shoulder and was not able to move over 30°. After ESPB, the patient was able to move her arm at the shoulder to approximately to 70° abduction without pain. She had acromioplasty and fibrous tissue resection in the shoulder joint including shoulder cap. In the PACU, her pain score was 8/10 on NRS, so she was given 50 µg fentanyl. Her PCA tramadol consumption was 240 mg in 24 hours. Despite the rescue analgesia treatment (IM diclofenac sodium, 75 mg and IV meperidine, 50 mg), her level of pain remained constant between 4/10 to 6/10 on NRS.

## Discussion

Shoulder surgeries, especially arthroscopic procedures, cause significant postoperative pain in the first 24 hours following surgery [6]. The interscalene block [5], suprascapular nerve block with or without axillary nerve block, and supraclavicular block may be used depending on the type of surgery [7]. ESPB has been reported for use in thoracic surgery [8], total hip arthroplasty [9], breast implant surgery [10], and chronic shoulder pain [4]. 

Previous examinations of cadavers with radiographic evidence have demonstrated that local anesthetic penetration develops through connective tissues and ligaments towards the final paravertebral area when it is injected deep (anterior) into the erector spinae muscle [3-4]. When it was applied at the level of T2 or T3, it was proposed to block C5 and C6 nerve roots including the suprascapular, axillary, and the lateral pectoral nerves. The blockage of these nerves may result in a sensory block at the shoulder joint, although the cap of the shoulder joint is innervated from the superior cervical plexus (C3-C4). The effect of the ESPB may vary according to anatomical variations, volume, and other factors which may change the spread of the local anesthetics in the cervical plexus [7]. The supraclavicular nerve, which accepts innervation from C3 and C5 and gives cutaneous innervation to the anterior and posterior supraclavicular area, might not be covered by ESPB. Therefore, its blockage should be considered carefully in shoulder surgeries. The humeral bone gets innervation from the axillary nerve (C5, C6), long thoracic nerve (C5, C6, C7), and the suprascapular sensory blockade of the area innervated by these nerves [11]. Blockage of the C5, C6, and C7 nerve roots with ESPB can, therefore, be accomplished. Its main effect is on the brachial plexus which may obtain adequate analgesia and was not observed in any of our patients. Complications associated with regional interventions for postoperative analgesia in shoulder surgery may include a motor block of the hand, arm, or both. In case of signs of loss of grip power, the patient may ignore the absence of pain [5]. Before surgery commenced, all patients described acute relief of their pain with ESPB, and painless range of motion in the shoulder joint immediately increased above their resting level range of motion. This confirms that while an effective and successful block was applied in the second case, effective postoperative analgesia did not reach an adequate level.

The intensity of the postoperative pain is related to the extent of the surgical intervention and tissue damage created by the extent of adhesiolysis [6]. Rotator cuff repair and debridement of damaged tissue may also cause intense pain. The level of existing damage, immobility, and restrictions in the shoulder joint may necessitate major surgical intervention. Additionally, the glenohumeral joint capsule or the acromioclavicular joint capsule innervation may vary individually such that any surgical procedures including the shoulder cap may require a cervical plexus blockade [5]. All these factors may influence distinct postoperative pain scores recorded in the three different shoulder surgery operations presented here. The higher pain scores in the second case might be related to the damage of the shoulder cap which gets innervation from the superior cervical plexus (C3-C4) and may be spared in ESPB. Unfortunately, in the postoperative period, assessment of sensory loss over the cap of the shoulder and clavicle were not possible due to the sterile closure of the wound.

## Conclusions

There are no examples or guidelines for the use of ESPB for the treatment of postoperative pain in shoulder surgeries in the literature. The surgery type, varying concentrations of local anesthesia, volumes, and individual anatomical variations may result in different outcomes. Further randomised controlled clinical trials in shoulder surgeries may reveal the analgesic range, opioid-sparing effect, and ideal dose concentration of ESPB, along with the possible drawbacks.
